# GIN-TONIC: non-hierarchical full-text indexing for graph genomes

**DOI:** 10.1093/nargab/lqae159

**Published:** 2024-12-11

**Authors:** Ünsal Öztürk, Marco Mattavelli, Paolo Ribeca

**Affiliations:** SCI-STI-MM, EPFL, ELB 118, Station 11, 1015, Lausanne, Switzerland; SCI-STI-MM, EPFL, ELB 118, Station 11, 1015, Lausanne, Switzerland; Biomathematics and Statistics Scotland, The James Hutton Institute, Peter Guthrie Tait Road, EH9 3FD, Edinburgh, United Kingdom; Clinical and Emerging Infection, UK Health Security Agency, 61 Colindale Avenue, NW9 5EQ, London, United Kingdom; NIHR Health Protection Research Unit in Genomics and Enabling Data, University of Warwick, Gibbet Hill Road, CV4 7AL, Coventry, United Kingdom; NIHR Health Protection Research Unit in Gastrointestinal Infections, University of Liverpool, 8 West Derby Street, L69 7BE, Liverpool, United Kingdom

## Abstract

This paper presents a new data structure, GIN-TONIC (**G**raph **IN**dexing **T**hrough **O**ptimal **N**ear **I**nterval **C**ompaction), designed to index arbitrary string-labelled directed graphs representing, for instance, pangenomes or transcriptomes. GIN-TONIC provides several capabilities not offered by other graph-indexing methods based on the FM-Index. It is non-hierarchical, handling a graph as a monolithic object; it indexes at nucleotide resolution all possible walks in the graph without the need to explicitly store them; it supports exact substring queries in polynomial time and space for all possible walk roots in the graph, even if there are exponentially many walks corresponding to such roots. Specific ad-hoc optimizations, such as precomputed caches, allow GIN-TONIC to achieve excellent performance for input graphs of various topologies and sizes. Robust scalability capabilities and a querying performance close to that of a linear FM-Index are demonstrated for two real-world applications on the scale of human pangenomes and transcriptomes. Source code and associated benchmarks are available on GitHub.

## Introduction

The importance of being able to represent DNA sequences as complex graphs rather than linear text is well acknowledged in the literature, with increasing attention being given to pangenome-based applications due to their advantages in natively representing variants or alternate sequences (1–4). Another relevant use case is transcriptomics, where the possible space of spliced sequences can be fully and directly represented by a graph (5). Whereas indexing text has been widely studied in literature, the problem of fully indexing string-labelled graph is more difficult and still an open one.

Due to its efficiency in matching queries against large linear reference sequences (6–8), the FM-Index (9) is a data structure widely used in bioinformatics. Although implementations of the FM-Index abound, each one striking a different balance between compression and speed (10,11), a common use for them is to check the existence, and compute the locations, of the exact matches of shorter *k*-mers from a query sequence. This process, often referred to as seeding, is a common procedure in alignment software (12). Here, we develop an efficient solution to extend this capability to graph genomes, which cannot be represented by the regular FM-Index.

Aligning reads to graph genomes and pangenomes offers a more comprehensive view of genomic diversity. However, efficiently indexing these structures is challenging—unlike linear sequences, graphs can contain complex looping structures and multiple alternative walks, making indexing more difficult (1,13) due to the combinatorial nature of the number of walks to be explored (14,15). An ideal, efficient graph indexing data structure should keep the same good qualities of the FM-Index—notably, the ability to return all the exact matches in polynomial time—while also being able to transparently explore all matches along all possible graph walks.

Recent related work based on the Burrows-Wheeler Transform (BWT) (16) and the FM-Index includes the vg toolkit (17) and extensions of the BWT to the positional BWT (18) for pangenomic-level haplotype indexing (graph BWT, graph positional BWT) (19,20). Further developments add synthetic haplotypes or greedy graph covers to allow nucleotide-domain indexing (21). HISAT 2 and 3 (22,23) use a different, hierarchical approach consisting of a global FM-Index for the whole genome and local FM-Indices constructed over smaller genomic regions. GCSA2 from vg and PSI enumerate string paths on the input graph and approximate the initial graph with a de Bruijn graph, which is then indexed with a BWT-based technique (24,25). Unlike these tools, theoretical approaches to fully generalize the combinatorial properties of the BWT to special subclasses of character-labelled graphs have also been proposed. The concept of Wheeler graphs (26) extends the main properties of LF-mapping traversals, establishing properties analogous to those of the BWT, such as the query time being proportional to query length, under the assumption of path coherence and monotonicity. Strategies based on Wheeler graphs such as r-indexing (27), tunneling techniques (28) and prefix-free parsing (29) improve the theoretical indexing performance of Wheeler graphs. Further generalizations are made in (30), in which the authors describe a family of transformations that support matching in FM-Index-like time. A different approach is followed in (31–33) by considering a relaxation of Wheeler orders to co-lex orders or arbitrary relations, which allows pattern matching on the graph in polynomial time (34). However, due the computational difficulty in even recognizing Wheeler graphs, which is NP-Complete, the scaling to large graph instances remains an algorithmic and practical challenge, rendering algorithmic frameworks based on Wheeler graphs impractical for human-scale pangenomics (15,35).

Here, we introduce a novel graph indexing approach called GIN-TONIC (**G**raph **IN**dexing **T**hrough **O**ptimal **N**ear **I**nterval **C**ompaction). It is designed to handle string-labelled directed graphs of arbitrary topology by indexing all possible string walks without explicitly storing them. Crucially, it allows for efficient exact lookups of substring queries of *unrestricted length* in polynomial time and space (excluding the size of the output, which may be exponential); it does not require the construction of multiple indices or explicit indexing of enumerated walks, and it easily scales up to the size of human (pan)genomes and transcriptomes.

GIN-TONIC constructs a special encoding of the graph (similar to the one in (36)) and efficiently traverses it in the suffix array domain with the help of an FM-Index, allowing for an exact substring matching mechanism with quadratic complexity in the length of the query *Q* and number of graph vertices *V*, with a worst-case time complexity of *O*(|*Q*|^2^|*V*|^2^log |*Q*||*V*| + *k*) in *O*(|*Q*||*V*| + *k*) space per query, where *k* is the number of occurrences of the query in the graph. This work also describes how such complexity can be further improved by approximating a solution to an NP-Hard problem. The index itself can be constructed in *O*((|*V*| + |*E*|)log |*V*| + |∑_*i*_*S*_*i*_|) time, where ∑_*i*_|*S*_*i*_| is the total length of the string labels and |*E*| is the number of edges. The space requirement depends on the FM-Index implementation and could range from compressed to expansive, but never exceeds *O*(|*V*|^2^ + |∑_*i*_*S*_*i*_|).

Finally, we describe and benchmark an implementation of GIN-TONIC. Thanks to the addition of a suitable caching mechanism, GIN-TONIC is able to offer excellent performance despite having reasonable memory requirements.

## Materials and methods

### Notations

**Strings**. A string of length *L* over the finite alphabet Σ is defined as a sequence of characters *S* = *s*_0_*s*_1_...*s*_*L* − 1_ with *s*_*i*_ ∈ Σ ∀*i* ∈ {0, ..., *L* − 1}, where *L* = |*S*| is the length of the string. We denote the set of all strings over the alphabet Σ as Σ*, the set of all strings of length *L* as Σ^*L*^, and the set of all strings up to (and including) length *L* as Σ^≤*L*^. Strings are indexed starting from 0, and the *j*th character of a string *S* is denoted as *S*[*j* − 1]. The rank operation over a string *S* is defined as $\small {Rank}(S,\sigma ,i)= \sum _{j=0}^{i} \mathbb {1}\lbrace S[j] = \sigma \rbrace$ where σ ∈ Σ, and 0 ≤ *i* < |*S*|. A substring of a string *S* over the half open character range [*a*, *b*) is denoted as *S*[*a*: *b*] and defined as *s*_*a*_...*s*_*b* − 1_ for *b* > *a*, $|S|> a{\color {red} \ge }0$, and |*S*| ≥ *b* > 0; it is taken to be *S*[*a*: *b*] = ε otherwise. ⊕ denotes iterative string concatenation, and defined to be $\oplus _{i={1}}^{N} S_i = S_1...S_N$ similar to the summation operator. $\mathcal {B}(S)$ is the BWT of *S*.


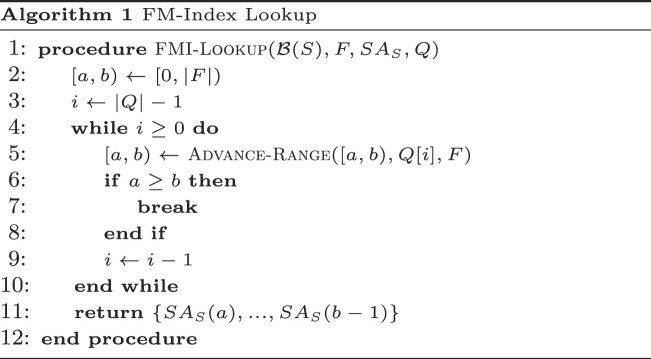


**FM-Index lookup**. Given some string query *Q*, the FM-Index of a string *S* returns the location of all indices *i*_0_, ..., *i*_*m* − 1_ such that *Q* occurs as a substring of *S* at *i*_*j*_ for *j* ∈ {0, ..., *m* − 1} through variations of Algorithm 1. The subroutine $\small {Advance-Range}$ takes as input a half-open range over the suffix array and uses the *LF*-mapping to get a progressively refined range by walking through all the characters in the query.

**String graphs**. A string graph $G(V,E,\mathbb {S})$ is defined as a set of vertices *V* = {*V*_1_, ..., *V*_*N*_} labelled by non-empty strings $\mathbb {S}=\lbrace S_1, ..., S_N \rbrace$ such that *S*_*i*_ is the label of *V*_*i*_, and a set of directed edges *E* = {(*V*_*i*_, *V*_*j*_)∣*V*_*i*_, *V*_*j*_ ∈ *V*}. The set of incoming neighbors for a vertex *V*_*i*_ is denoted as $\mathcal {N}^{-}(i)$. A string *S* is said to be induced or encoded by a string graph $G(V,E,\mathbb {S})$ if there exists a walk $W=V_{i_1} \rightarrow ... \rightarrow V_{i_w}$ on *G* such that *S* is a substring of $S_W = S_{i_1}...S_{i_w}$. Conversely, the strings induced by a walk *W* are defined to be the set of all substrings of *S*_*W*_.

### Problem definition

The main problem of interest consists of enumerating all the starting points (‘roots’) from which there exist walks inducing the given query, i.e.:

Definition 1.

Let $G(V,E,\mathbb {S})$ be a string graph, and let *Q* be a non-empty string over a non-empty alphabet Σ. We would like to enumerate all vertex-offset tuples (*V*_*i*_, *o*_*i*_), where *V*_*i*_ ∈ *V* and $o_i \in \mathbb {N}$ is an offset into the label of *V*_*i*_, such that there exists a walk $W=V_{a_1}\rightarrow ... \rightarrow V_{a_w}$ for some sequence of vertex indices *a*_*j*_ with the label concatenation $S_W = \oplus _{j=0}^w S_{a_j} = S_{a_1}...S_{a_w}$ satisfying the following:

- (The root of the walk is *V*_*i*_) $V_{a_1}=V_i$.
- (The walk induces *Q*) *Q* = *S*_*W*_[*o*_*i*_: *o*_*j*_] for some *o*_*j*_.
- (The walk induces *Q* minimally) $|S_{a_1}| > o_i \ge 0$ and ${\sum \nolimits_{k=1}^w}|S_{a_k}|\ge o_j > {\sum \nolimits_{k=1}^{w-1}}|S_{a_{k}}|$.

We define three types of queries, which return:

- Find: Suffix array ranges corresponding to walk roots
- Locate: Walk roots in the graph domain as (*V*_*i*_, *o*_*i*_)
- Walk: Reconstructed walks rooted at (*V*_*i*_, *o*_*i*_)

**Figure 1.**
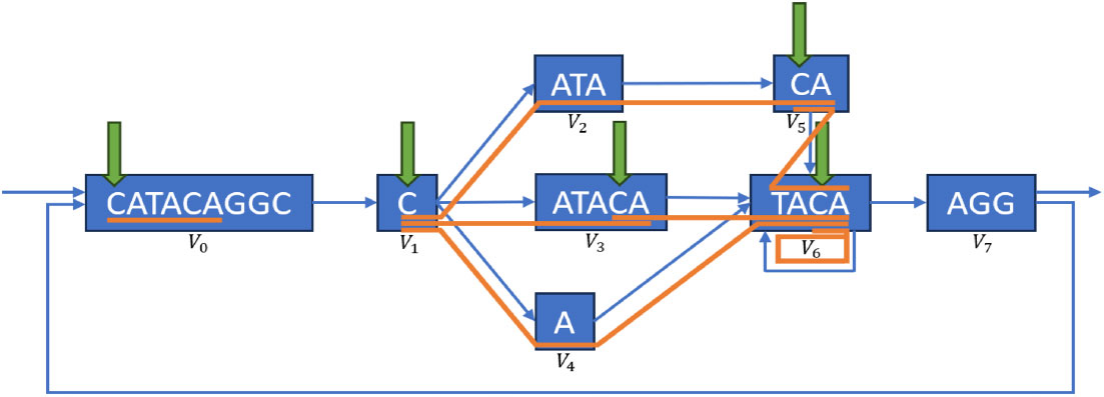
Example segment of a complex region of a string graph. Vertices are denoted with rectangles, and edges with arrows, and vertex labels as text inside rectangles representing vertices. Walk roots corresponding to the string CATACA—equivalent to vertex index and offsets—are marked with vertical arrows pointing to vertex labels. The walks inducing CATACA starting from the marked walk roots are underlined. Note that one root may induce multiple walks of the example string, that walks stemming from different roots may partially overlap, and that the same path can contain more than one match. Locate queries output all walk roots (vertical arrows), and Walk queries output all paths (segments underlining vertex labels).

Locate queries first call Find queries to find suffix array ranges, and Walk queries call Locate queries to start traversing the graph from walk roots. Walks and walk roots are visualized in Figure 1.

### GIN-TONIC

This section defines the data structures used by GIN-TONIC.

Definition 2.

**GIN-TONIC** is a data structure defined as a 4-tuple $(\small {I}_{\small {FMI}}(G,\Pi ), R, r_{\sigma _0}, \mathcal {C})$ where:

-
$\Pi : \mathbb {N} \rightarrow \mathbb {N}$ is a permutation over the set of vertices of a string graph *G*.
-
$\small {I}_{\small {FMI}}$ is an FM-Index constructed over a *graph encoding*$\small {GE}(G,\Pi )$.
-
$r_{\sigma _0}$ is a lookup table translating ranks of a special character σ_0_ in the Burrows-Wheeler domain of the graph encoding $\small {GE}(G,\Pi )$ to corresponding ranks in the text domain.
- *R* is a data structure mapping ranges of a special character σ_0_ to ranges of σ_1_ based on *E*, over $\small {SA}_{\small {GE}(G,\Pi )}$.
-
$\mathcal {C}$ is an optional, pre-computed cache of suffix array ranges corresponding to all substrings of length 1...*d* induced by the graph.

Definitions for $\small {GE}(G,\Pi )$, $r_{\sigma _0}$, *R*, and *C*, follow.

### The graph encoding

The graph encoding $\small {GE}(G,\Pi )$ of a string graph *G* over a permutation Π is a string using special reserved characters to encode information on the permutation Π, the string labels $S_i\in \mathbb {S}$ of *G*, and the mapping between the indices of vertices *V* of *G* and the string labels of *G*. The query algorithm will use the special characters to jump to other vertices when necessary.

Definition 3.

The **graph encoding of a string graph** is defined as the following string concatenation:


(1) \begin{eqnarray*} \small {GE}(G(V,E,\mathbb {S}), \Pi ) = \sigma _0 S_1 \sigma _1 A_1 \sigma _0 S_2 \sigma _1 A_2 ... \sigma _0 S_N \sigma _1 A_N \epsilon \end{eqnarray*}

(2) \begin{eqnarray*} = \left(\oplus _{i=1}^N \left( \sigma _0 S_i \sigma _1 A_i \right)\right) \epsilon \end{eqnarray*}

where Π denotes some permutation from {1, ..., *N*} to {1, ..., *N*}, $S_i \in {\mathbb S}$ are strings over the string label alphabet Σ; σ_0_, σ_1_∉Σ are two special characters, respectively, indicating the start and end of the string label *S*_*i*_ associated with each vertex *V*_*i*_, and *A*_*i*_ are strings over another alphabet Σ_Π_ such that Σ∩Σ_Π_ = ∅ and σ_0_, σ_1_∉Σ_Π_, and the lexical order of *A*_*i*_ depends on Π. The strings *A*_*i*_ are discussed in the section ‘The vertex permutation’. Hence, the alphabet of string $\small {GE}(G,\Pi )$ is Σ∪Σ_Π_∪{σ_0_, σ_1_}. The lexicographic ordering of the alphabet of $\small {GE}(G,\Pi )$ is defined as $\sigma _0 < \sigma _1 < c_{\Sigma _{\Pi }} < c_{\Sigma }$ for any $c_{\Sigma _{\Pi }} \in \Sigma _{\Pi }, c_{\Sigma } \in \Sigma$.

Positionally associating each vertex label with a σ_0_ and querying its rank allows one to obtain the index of the vertex in the text domain. When traversing the LF-mapping of the graph encoding, encountering a non-empty range of σ_0_ indicates that the current match has exhausted one or more vertex labels starting from which the currently matches suffix is induced, and the matching process might succeed by considering incoming neighbors if there are any. This property can be exploited to index all walks on the graph.

### The suffix array range translator

The range translator *R* encodes the connectivity of *G* defined by *E* in the BW-domain. This is achieved by associating ranges of σ_0_, which express edge destinations, to ranges of σ_1_, which express edge sources. The query algorithm uses this mapping to traverse multiple vertices whenever necessary during matching.

Definition 4.

Given two maps $r_{\sigma _0}$, $r_{\sigma _1}$ that can be computed from $\small {SA}_{\small {GE}(G,\Pi )}$, with $r_{\sigma _0}$ mapping ranks of σ_0_ from $\mathcal {B}(\small {GE}(G,\Pi ))$ to $\small {GE}(G,\Pi )$, and $r_{\sigma _1}$ mapping the ranks of σ_1_ from $\small {GE}(G,\Pi )$ to $\mathcal {B}(\small {GE}(G,\Pi ))$, the **single-vertex range translator**$R_{\sigma _0}:{\mathbb N}\rightarrow 2^{\mathbb {N}}$ is defined as:


(3) \begin{eqnarray*} R_{\sigma _0}(i) = \bigcup _{k\in \mathcal {N^{-}}(r_{\sigma _0}(i))} r_{\sigma _1}(k). \end{eqnarray*}

$R_{\sigma _0}$ expresses as ranges of σ_1_ in $\small {SA}_{\small {GE}(G,\Pi )}$ the notion of incoming neighbours of a vertex marked with a σ_0_. The **range translator**$R:{\mathbb I}\rightarrow 2^{\mathbb {I}}$, where ${\mathbb I}=\lbrace [a,b] |$$a,b\in {\mathbb N}, a\le b\rbrace$, is defined as:


(4) \begin{eqnarray*} R([a,b]) = C_{\mathbb I}\left(\bigcup _{i=a}^{b} R_{\sigma _0}(i)\right) = C_{\mathbb I}\left(\bigcup _{i=a}^{b} \left( \bigcup _{k\in \mathcal {N^{-}}(r_{\sigma _0}(i))} r_{\sigma _1}(k)\right)\right) \end{eqnarray*}

where $C_{\mathbb I}:2^{\mathbb N}\rightarrow 2^{\mathbb I}$ is a function compacting a set of natural numbers into a set of intervals. *R* expresses the incoming neighbors of a range of vertices in $\small {SA}_{\small {GE}(G,\Pi )}$.

We implement *R* in GIN-TONIC as a binary tree where each node contains a range key [*a*, *b*] and a list of intervals as values, called the interval-merge tree (IMT). *R* can be computed in Θ(|*V*|log |*V*|) time with *O*(|*V*|) space through this tree, which is defined as follows:

- Each node (denoted with *T*) is associated with two children nodes, one range, and an interval list.
- A parent node with a range [*a*, *b*] splits its range between its two children: the left child has the key-range $\left[ a, \left\lfloor \frac{a + b}{2} \right\rfloor \right]$, the right child has $\left[ \left\lfloor \frac{a + b}{2} + 1 \right\rfloor , b \right]$.
- Leaf nodes have key range [*k*, *k*], and interval list $R_{\sigma _{0}}(k)$.
- A parent with children interval lists ν_*l*_, ν_*r*_ has a merged and compacted list of ν_*l*_ and ν_*r*_.

*R* can be evaluated on [*a*, *b*] by querying the tree recursively, as per Algorithm 2. Alternatively, implementations can merge references from all lists using a priority queue for a k-way merge.


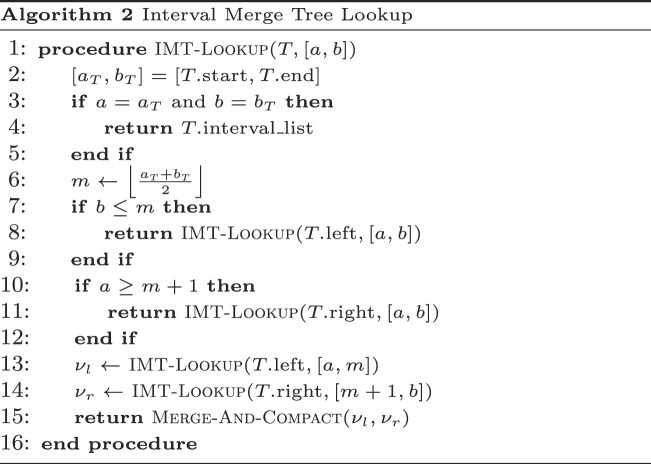


### The vertex permutation

The permutation Π in $\small {GE}(G,\Pi )$ is used to generate a string family *A*_*i*_ where each *A*_*i*_ follows each σ_1_ tied to vertex *i* according to Equation 1. This permutation dictates the order of σ_1_s in ${\mathcal B}(\small {GE}(G,\Pi ))$; it allows to permute σ_1_s independent of the encoding and, possibly, to make suffix array ranges of walks that share a prefix consecutive, which increases the efficiency of querying. Π can be enforced by selecting a unique alphabet Σ_Π_, distinct from Σ∪{σ_0_, σ_1_}, generating *N* = |*V*| strings of length $\lceil \log _{|\Sigma _{\Pi }|}(N)\rceil$, and arranging them lexicographically. Denote these sorted strings as *X*_1_, ..., *X*_*N*_. Given any σ_1_, appending an *X*_*i*_ to it forces its rank in ${\mathcal B}(\small {GE}(G,\Pi ))$ to be *i*. Hence, setting $X_{\Pi ^{-1}(i)}=A_{i}$ ensures that the σ_1_ with rank *i* in $\small {GE}(G,\Pi )$ has rank Π(*i*) in $\mathcal {B}(\small {GE}(G,\Pi ))$. Generally, using any sorted set of |Σ_Π_|-ary uniquely decodable codes for *X*_*i*_ achieves the same result with reduced overhead.

One can choose the permutation Π so that ranges of σ_1_s corresponding to the starting vertices of walks with a shared prefix are consecutive in the suffix array; that will result in faster query times. However, determining an optimally consecutive permutation is an NP-complete problem. Define


(5) \begin{eqnarray*} \mathcal {V}_Q = \left\lbrace V_{i_0} | \exists W=V_{i_0}\rightarrow ...\in \mathcal {W} \wedge S_W[:\left|Q\right|] = Q \right\rbrace \end{eqnarray*}

as the root of walks containing *Q*. To minimize interval counts returned by *R* for encountered ranges of σ_0_s during a query *Q*, define a constraint set *C*_*Q*_ for a query as:


(6) \begin{eqnarray*} C_Q = \bigcup _{V_i\in \mathcal {V}_Q} \bigcup _{j \in \mathcal {N}^-(i)}j \end{eqnarray*}

Choosing Π for all possible *Q* over all *C*_*Q*_s is equivalent to solving the Consecutive Block Minimization problem on a binary matrix *B* of size Σ^|*Q*|^ × |*V*| where each row corresponds to a query string *Q*, and for every *Q*_*i*_ at row *i*, the bit *B*[*i*][*j*] is set for all $j\in C_{Q_i}$. This yields an optimal Π, at the price of solving an NP-Hard but 1.5-approximable problem as shown in (37).

### Querying and construction algorithms

This section provides two algorithms to query and construct GIN-TONIC, based on the definitions and observations provided and discussed in the section ‘GIN-TONIC’.

Algorithm 3 defines the construction of an GIN-TONIC from a string graph *G*, a permutation Π, and its associated *X*_*i*_. The procedure initially generates the graph encoding, calculates its suffix array and sorts the sub-array of suffix rank-to-offset mappings for σ_0_s to derive the $r_{\sigma _0},r_{\sigma _1}$. It then constructs an FM-Index using the suffix array. The overall computational complexity is *O*((|*V*| + |*E*|)log |*V*| + ∑_*i*_|*S*_*i*_|).


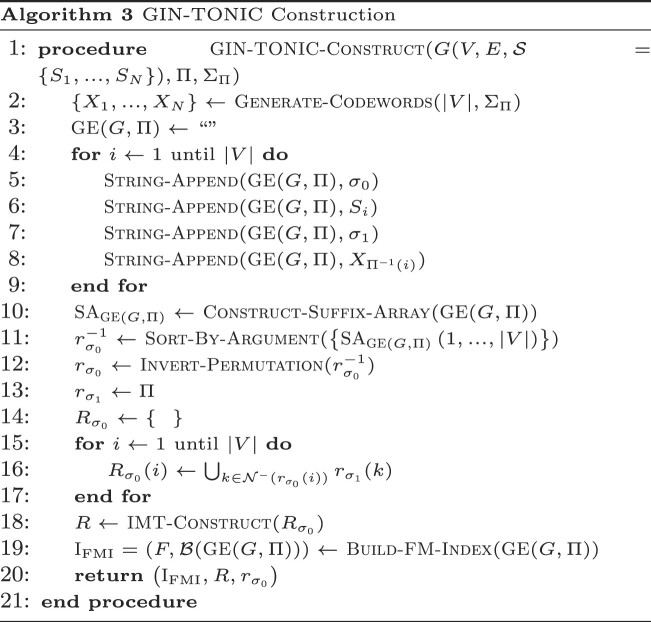



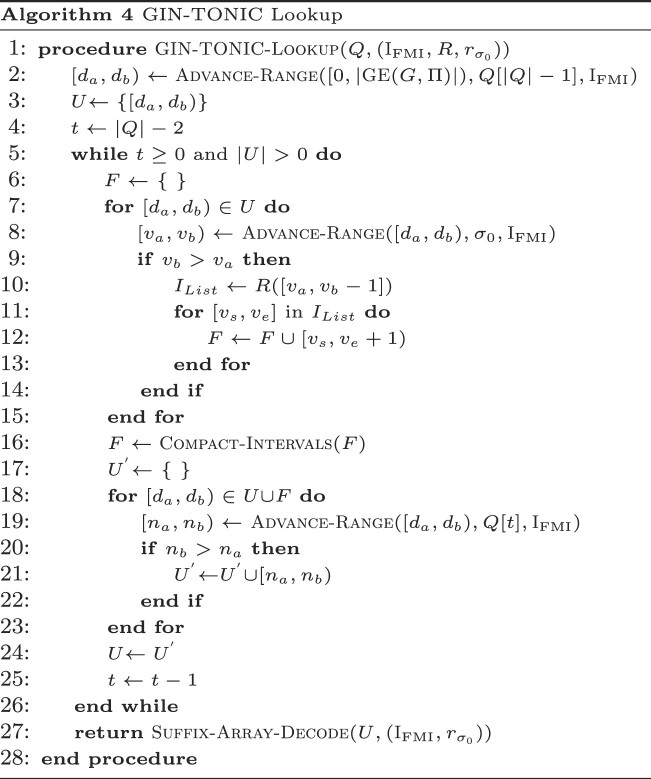


Algorithm 4 implements Locate queries, and returning *U* at line 27 instead of the decoded SA implements Find queries. Initially, the algorithm matches a query character to the graph encoding by updating the suffix array range based on the last character of the query over the full LF-mapping of $\small {GE}(G,\Pi )$ in lines 2−4. This range then becomes the initial range to be tracked. Subsequently, the algorithm undergoes a loop, executing the ensuing steps for |*Q*| − 1 iterations:

- Forking Phase (lines 6−16): For each tracked range, the algorithm assesses if the character σ_0_ is a preceding character in the LF-mapping of $\small {GE}(G,\Pi )$. If so, then some walks must contain the ongoing partial match as a prefix (verified in lines 8−9). The algorithm then fetches the ranges of incoming neighbors using the range translator *R*. Each range from *R* is termed a ‘fork’, and such forks are collected in a separate set *F* (line 12).
- Compaction Phase (line 16): The forks from *R* represent suffix array ranges over σ_1_. Although many forks might be retrieved, they can be compacted into fewer intervals. This phase determines the union of these intervals and simplifies their representation using the subroutine $\small {Compact-Intervals}$. Assuming *R* returns sorted ranges, this operation is linear in the number of intervals.
- Advance Phase (lines 17−25): For each tracked range and fork, the algorithm advances to the next query character through the Advance-Range procedure on the FM-Index. A separate set ${U}^{^{\prime }}$ filters out ranges without the next character’s occurrence in the LF-Mapping. The algorithm then updates its tracked ranges to ${U}^{^{\prime }}$ and decrements the counter *t*, which indicates the next character position in *Q* to be considered.

**Figure 2.**
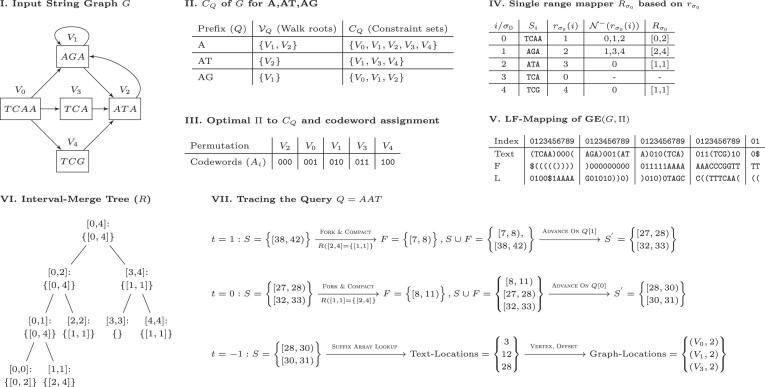
Illustrated examples of how to build and query a GIN-TONIC. (**I**) A string graph of five vertices and eight edges. (**II**) Arbitrarily sampled constraint sets for three string label prefixes *A*, *AT*, *AG* generated according to the procedure in the section ‘The vertex permutation’. (**III**) Codeword assignment to enforce a permutation preserving a consecutive order of the constraint sets in (II). (**IV**) The single-vertex range translator over σ_0_. $R_{\sigma _0}$ contains the ranges of $\mathcal {N}^-(r_{\sigma _0}(i))$ in the permutation given in (III). (**V**) LF-Mapping constructed on the graph encoding with the permutation obtained in (III) with $\sigma _0=\texttt {(}$ and $\sigma _1=\texttt {)}$. The LF-Mapping is partitioned into chunks of 10 characters for visual and algorithm tracing convenience. (**VI**) IMT based on the single range mapper $R_{\sigma _0}$. (**VII**) Matching *Q* = *AAT* using the data structures in V and VI. The query is successively extended one character at a time starting from its end to the beginning as in the linear FM-Index.

After loop termination, *U* contains the suffix array ranges that represent the starting points of walks inducing *Q*. These results are converted to the text domain using Suffix-Array-Decode. The algorithm’s time complexity is determined by the evaluation frequency of *R*, Advance-Range and Compact-Intervals, and the range count returned by *R*. It can be demonstrated that at iteration *t*, the algorithm holds at most (*t* − 1)⌈|*V*|/2⌉ intervals in the worst case. This is because Compact-Intervals never returns more than ⌈|*V*|/2⌉ intervals by the pigeonhole principle. This leads to a worst-case time complexity of *O*(|*Q*|^2^|*V*|^2^log |*Q*||*V*| + *k*), where *k* is the number of walk roots from which the query can be reconstructed. However, if Π is optimal, producing the fewest possible number of runs in *B* for every prefix, then Compact-Intervals outputs the fewest number of intervals. Consequently, the worst-case time complexity of the algorithm may be improved depending on the number of runs in the optimal *B* yielded by the graph topology. Note that the given bounds are pessimistic, and only occur when $\small {GE}$ is adversarially constructed in order to maximize the number of intervals returned by *R*. Typically, the time complexity depends on the number of occurrences in the graph of walk roots corresponding to the query, i.e. the size of the output. For rigorous mathematical derivations of the bounds on the number of ranges returned and worst-case time complexity of querying, see the section ‘Asymptotic and Practical Time Complexity of GIN-TONIC Lookup’ in the Supplementary Material (Supplementary Figure S4), and Figure 2 illustrates how the algorithm works on example inputs.

### The pre-computed suffix array range cache

In general, the initial iterations of a query yield disjoint suffix array ranges returned through *R*, most of which are subsequently discarded during the algorithm’s advance phase, leading to unnecessary computation. A suffix array cache $\mathcal {C}$ with depth *d* is defined as a function $\mathcal {C}:\Sigma ^{\le d} \rightarrow 2^{\mathbb I}$ which takes a string argument *Q* with length at most *d* and returns the suffix array ranges containing walks inducing *Q*. By looking up $\mathcal {C}$ and initializing *U* at line 3 of Algorithm 4, then iterating only *t* − *d* times, we can significantly cut down redundant computations from earlier iterations, albeit with increased storage demands. While one could represent $\mathcal {C}$ with a hash table, its static nature allows for a compressed FM-Index implementation. Given unique string keys *S*_1_, ..., *S*_*K*_ ∈ Σ^≤*d*^ and their related suffix array interval lists *I*_1_, ..., *I*_*K*_, we can define the cache encoding as $\mathcal {C}\text{E}(G,\Pi ) = (\oplus _{i=1}^{K}\sigma S_i)\sigma \epsilon$, where σ∉Σ is a unique marker. In $\mathcal {C}\text{E}$, the rank of σ matches the string key index. The interval lists are indexed using an FM-Index over $\mathcal {C}\text{E}$, and the lists are permuted as $I_{\Pi _{\text{SA}(1)}},...,I_{\Pi _{\text{SA}(K)}}$, with Π_SA(*i*)_ indicating the permutation of σs in $\mathcal {B}(\mathcal {C}\text{E})$. To look up this index for a string *Q*, one matches σ*Q*σ against the FM-Index and navigates to the interval list $I_{\Pi _{\text{SA}(r)}}$, where *r* is the rank of σ after matching σ*Q*σ. Due to its structure, the FM-Index will have at most one match for σ*Q*σ. $\mathcal {C}$ enhances query efficiency considerably as discussed in the following section. The advantage of having this cache compared to a table is its ability to represent keys (i.e., all Σ^≤*d*^ appearing in the graph) in compressed form while maintaining considerable querying speeds.

## Experiments and results

### Benchmark setup

Performance of the proposed algorithms was assessed on two use cases. The first one involves a human pangenome graph, constructed from 20 haplotype-collapsed assemblies as described in (38), with 148 618 vertices, 214 995 edges and a total label length of 3.14 × 10^9^ base pairs. The second use case considers the splicing graph of a human transcriptome with 1 029 466 vertices, 1 523 321 edges and a total label length of 6.5 × 10^9^ base pairs. This was generated by projecting the annotations of transcripts from GENCODE Release 40 (39) onto the human reference genome GRCh38.p13, with a custom awk script available from (40). Forward and reverse strands were considered separately in the same file. Additional statistics for the two graphs are presented in Supplementary Table S1. Experiments were conducted utilising a single thread on a machine with an AMD Ryzen Threadripper 3990X running at 2.2 GHz, 256 GB DDR4 RAM clocked at 2.7 GHz and a 1 TB SSD with maximum read/write speeds of 7.0/5.0 GB/s. For a larger graph, please see the section entitled ‘Scalability Tests with Multi-Indexing’ in the Supplementary Material (Supplementary Figures S5 and S6, and Supplementary Tables S3 and S4).

**Table 1.** Wall-clock index construction times, and sizes. (fast) and (native) refer to our FM-Index implementations, and (sdsl) to a compressed implementation using Huffman-shaped wavelet-trees on top of a particular bit-vector

Index Type	Pangenome		Transcriptome	
Name	Size (MB)	Time (s)	Size (MB)	Time (s)
(Native) **16**	12,800	649	26,700	1,760
(Native) **32**	7,360	612	15,400	1,600
(Native) **64**	4,640	570	9,720	1,570
(Fast) **32**	3,113	615	6,523	1,639
(**sdsl**) **Hybrid**	1,336	747	2,839	1,932
**(** **sdsl** ) **RRR**	1,278	777	2,716	2,012

**Table 2.** Average time taken in microseconds for Find queries and walk root decodes, and total walk root counts (#) for 65 536 adversarial queries over the input items with (fast)

Dataset	Pangenome			Transcriptome		
Query	Find	Decode	#	Find	Decode	#
Length	(μs)	(μs)		(μs)	(μs)	
**16**	16.62	1.22	516.65	9.19	1.47	297.29
**32**	43.37	1.12	46.61	16.81	1.10	9.69
**64**	52.57	1.49	3.92	24.35	1.01	1.05
**128**	71.42	2.17	1.52	43.11	0.78	1.01
**256**	82.71	2.26	1.19	65.63	0.50	1.00
**512**	110.16	1.45	1.15	111.97	0.38	1.00
**1024**	194.89	1.74	1.15	236.87	0.50	1.00
**2048**	385.78	2.19	1.15	447.11	0.62	1.00
**4096**	765.35	2.60	1.15	937.07	0.82	1.00

**Table 3.** Functional comparison with related work. Haplotype indices index paths expressed as sequences of pangenome segments. As opposed to exact matches, alignment identifies inexact matches showing sufficient similarity to the query according to some definition of string distance. Minimizer-based representations store a lookup table indexing minimisers extracted from the input sequences. Exhaustive searches return all existing matches, while heuristic searches can miss some. GCSA2 from vg searches for queries >256 bp can return more matches than those actually present

Method	What is indexed	Data representation	Query type	Query capability	Search type
gbwt/gbz (41) from vg	Haplotypes	Haplotypes	Haplotype match	Exact	Exhaustive
giraffe (21) from vg	Graph	Multiple	Graph alignment	Exact and inexact	Heuristic
minigraph (38)	Graph	Minimisers	Graph alignment	Exact and inexact	Heuristic
minimap2 (42)	Sequences	Minimisers	Text alignment	Exact and inexact	Heuristic
r-index (43)	Sequences	FM-Index	Text match	Exact	Exhaustive
MOVI (44)	Sequences	FM-Index	Text match	Exact	Exhaustive
GCSA2 (25) from vg	Graph	de Bruijn graph approximation	Graph match	Exact	Exhaustive up to 256 bp, false positives above
GIN-TONIC	Graph	Graph FM-Index	Graph match	Exact	Exhaustive

For each input item, permutations over the suffix array as described in the section ‘The vertex permutation’ were approximated for all walk prefixes of length 12 through simulated annealing; GIN-TONICs were then generated with a suffix array sampling period of 32. Construction times for all implementations including the SDSL-based ones, and resulting index sizes, are listed in Table 1. They are comparable to those of a linear FM-Index, as the length of $\small {GE}$ is approximately equal to the length of the human genome. To evaluate querying and decoding efficiency, 65 536 adversarial test queries maximizing the number of spanned vertices were sampled from the input file for each one of lengths 2^4^, ..., 2^12^. In addition, caches for each index with depths ranging from 1 to 12 were built as described in the section ‘The pre-computed suffix array range cache’ and summarized in Supplementary Table S2. Then, both our native and fast FM-Index implementations and two different implementations based on sdsl (45) (Huffman-shaped wavelet-trees with RRR bitvectors (11) and with hybrid bitvectors (46)) were benchmarked.

### Effective query length

While an FM-Index over a string traverses the LF-mapping exactly as many times as the length of the query *Q*, Algorithm 4 implies additional traversals every time the query needs to progress through the edges of the graph. This notion is captured by defining the *effective query length*$|Q|^{^{\prime }}$ as the number of LF-mappings done before the algorithm terminates, i.e., as the number of invocations of Advance-Range on line 20 of Algorithm 4. To quantify the overhead against the FM-Index, we also define the *relative effective query length* as:


(7) \begin{eqnarray*} \Delta (Q) \stackrel{\Delta }{=} \frac{|Q|^{^{\prime }} - |Q|}{|Q|} \end{eqnarray*}

Δ(*Q*) will be 0 for a linear genome and >1 for a graph genome, its precise value being fundamentally determined by the structure and complexity of the graph and the permutation. Notably, Δ(*Q*) is a pure factor independent of the actual FM-Index used by GIN-TONIC, and hence it is the same across all the implementations tested while benchmarking GIN-TONIC even though the times reported for each of them will differ. We experimentally determined that Δ(*Q*) is a legitimate metric when matches are not decoded, as Rank queries of Advance-Range turn out to be the computational bottleneck—in our benchmarks, evaluating *R* accounts for <1% of the execution time. Δ(*Q*) can be decreased by optimizing the permutation Π and/or increasing the cache depth *d*, which are explored in the following sections.

### Baseline benchmarks

**Figure 3.**
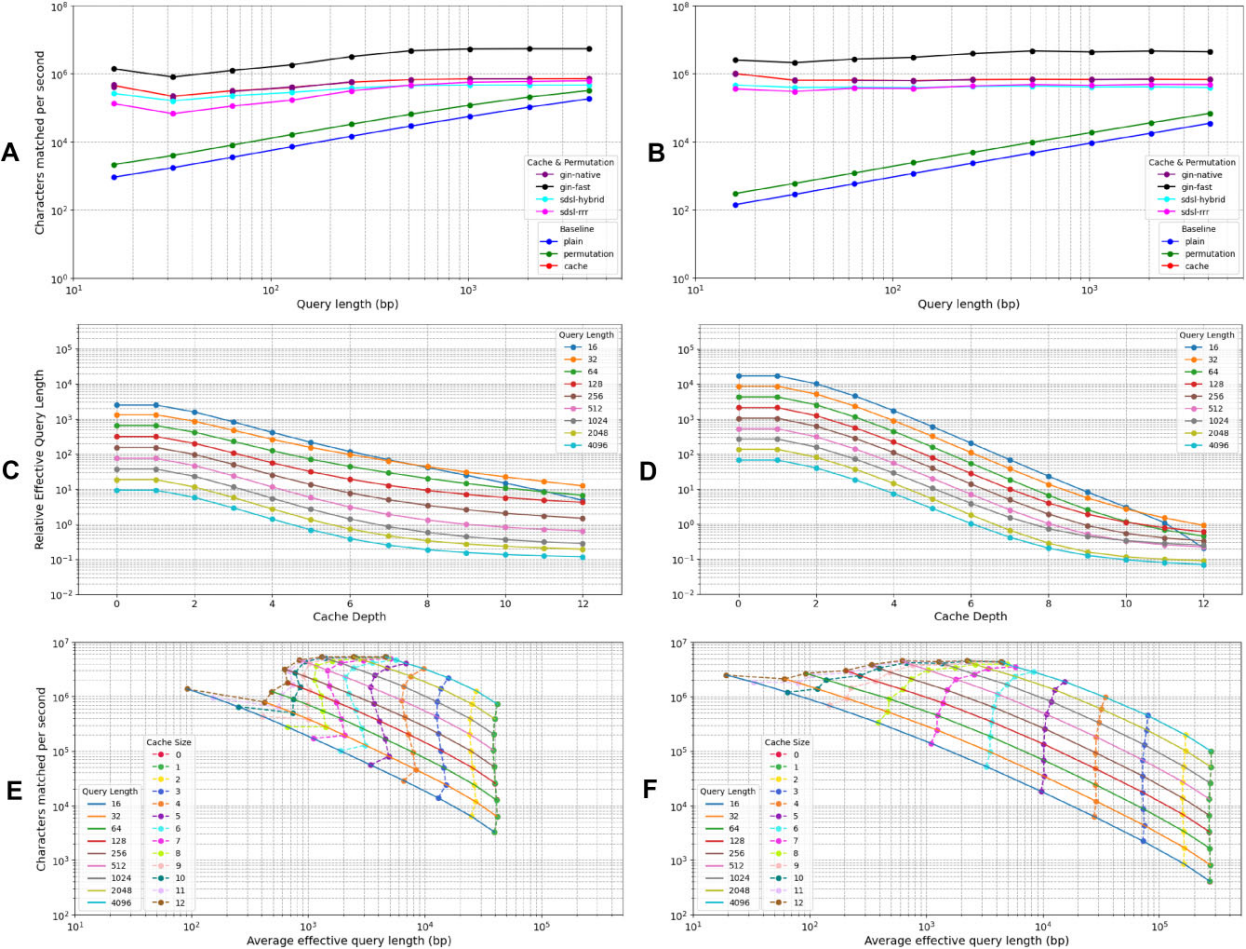
Benchmarking GIN-TONIC (fast) on adversarial queries. Left panels are for the pangenome, right panels for the transcriptome. Panels (**A**) and (**B**) show baseline performance using neither cache nor permutation, only permutation, only cache; and performance of different GIN-TONIC implementations (fast, native and sdsl-based) using both cache and permutation as characters matched per second. Panels (**C**) and (**D**) show average Δ(*Q*) as a function of cache depth. Caches of depth 0 and 1 have very similar performances close to the baseline since they cannot traverse more than one vertex, and hence they appear superimposed in the plots. Panels (**E**) and (**F**) show Δ(*Q*) versus characters matched per second, grouped by cache depth (dashed lines) and average effective query length (solid lines).

The main results of benchmarking GIN-TONIC’s baseline performance across different configurations (without optimizations; with optimized permutation; with cache; with optimized permutation and cache) are shown in Figure 3A and B. Data for the cache and permutation case are reported for four different FM-Index implementations (fast, native, sdsl with RRR bitvectors and sdsl with hybrid bitvectors).

Caches significantly outperform permutations in enhancing GIN-TONIC’s speed. An optimized permutation roughly doubled the effective query length and query throughput, whereas a cache led to several orders of magnitude of improvement (3 to 4 for short queries). However, using a permutation in addition to the cache can theoretically increase performance further; even though for our input items the gains were negligible. This is confirmed by Figure 3A and B—there is almost no discernible difference between the querying speeds obtained with just the cache and with gin-native, which uses both the permutation and a cache. The ‘Effect of Permutations’ section in the Supplementary Material andSupplementary Figures S1 and S2 contain more information regarding the impact of permutations.

In addition, the use of a cache effectively compensates for the complexity injected into the FM-Index by the graph structure. While without cache the number of characters matched per second by GIN-TONIC is not constant, unlike what happens with the FM-Index, that is not the case when a cache is used.

As for the effects of the implementation of the underlying full-text index, the ‘fast’ implementation of GIN-TONIC offers the best querying performance. It is built upon a cache-optimized DNA-based FM-Index designed to reduce alphabet sizes by not explicitly storing characters over which no rank queries are computed (i.e., string terminators, permutation encodings and σ_1_s), thus resulting in an effective alphabet size of 6 (A,C,G,T,N,σ_0_). Second in performance is our native FM-Index implementation, supporting arbitrary alphabet sizes but is not cache-optimized nor uses a reduced alphabet. These are followed by sdsl hybrid bitvectors and RRR bitvectors, which utilise a wavelet tree with compressed bitvectors; they are more compressive than our implementations but offer slower querying performance. The ‘fast’ GIN-TONIC implementation exceeds ∼10^6^ characters matched per second.

### Effect of the suffix array cache

Using a fixed permutation, cache depths from 1 to 12 were tested with rank and suffix array sampling rates set to 32; the results are illustrated in Figure 3C and D. They show that for both input graphs and for any given cache depth, Δ(*Q*) decreases as the query length increases; i.e., the longer the query, the closer the performance of GIN-TONIC was to that an FM-Index, and the more irrelevant the graph structure was. That can only happen if the number of jumps considered for longer queries decreases with the query length. We also observed that increasing the cache depth for a fixed query yielded several orders of magnitude of decrease in Δ(*Q*) for both input graphs, which indicates that a deeper cache is more able to optimize away the additional computation caused by the graph topology with respect to a linear FM-Index. Caches of order 10–12 make the values of Δ(*Q*) sufficiently close to 0 for GIN-TONIC to be practically usable.

### Effect of graph structure on querying speed

Strikingly, our benchmarks shine a light on the fundamental structural properties underlying the different graphs, as they directly determine the querying slowdown measured by Δ(*Q*).

Considering the actual performance of GIN-TONIC without a cache, one would expect the characters matched per second to be essentially independent of the query length starting from some query length on. After matching the first characters in the query, extending the query becomes cheap, thus rendering the work needed to match the rest of the query negligible. Hence, the expected curve showing Δ(*Q*) versus characters matched per second of GIN-TONIC without a cache over queries of increasing lengths is vertical. That can indeed be observed for both input graphs in Figure 3E and F. Note that points associated with caches of depth 0 and 1 coincide as they offer very similar, negligible improvements over the baseline.

For both the pangenome and transcriptome, increasing cache depth resulted in the transformation from a vertical curve, meaning that Δ(*Q*) was independent of the query length, to a horizontal curve, which means that the high cost for extending the first few characters in the query is eliminated by the cache, and the cost of extending the query by one character tends to become independent of the query length. Above certain cache depths, the number of characters matched per second starts to stabilize, implying that other operations of the algorithm have become the bottleneck.

However, there are differences between the pangenome and the transcriptome, as increasing the cache depth yields a flat curve for the transcriptome but not for the pangenome. This difference can be attributed to local graph structure. Vertices with shorter labels, high indegree and outdegree have the greatest potential to increase Δ(*Q*), as they will trigger more vertex traversals while extending the query. On the contrary, having a cache pre-computes the extensions of the query, and in the case where the label of a vertex is shorter than the depth of the cache, no additional computation arising from the topology of the graph is needed to extend the query.

This is confirmed experimentally by the local structure of pangenome and transcriptome. Supplementary Figure S3 shows the distribution of vertex label length versus vertex degree for each graph, and one can see that the distribution of vertex lengths in the pangenome is skewed towards longer lengths when compared to that for the transcriptome. In particular, a significant number of vertices of length ∼300 is present, which is probably an artifact pangenome construction; that is the likely reason why a flat curve is not achieved by a cache of depth 12. On the other hand, the vertex length distribution for the transcriptome is smooth and centered on much smaller values, which likely explains why the cache effectively decreases Δ(*Q*).

### Locate and Walk queries

Practical use of the index requires decoding suffix array ranges of matches *Q* into offsets of GE(*G*, Π). This decoding is independent of the internal details of Find queries, thus allowing query matching and suffix array range decoding into walk roots to be performed independently. In such a setting, the limiting factor in speed will be the slower of the two tasks.

Table 2 displays times spent performing Find queries of varying lengths and decoding the resulting suffix array ranges with the native implementation. For both inputs decoding was faster than Find for queries >16 bp, thus making Find the bottleneck. However, for shorter queries, given the higher number of matches returned, decoding was slower.

After decoding suffix array ranges, the offsets in GE(*G*, Π) are not yet in the format (*V*_*i*_, *o*_*i*_). By using a prefix-summed array of the offsets at which vertices start in GE(*G*, Π), index and offset of the vertex can be found efficiently in *O*(log *V*) time via binary search. This operation is fast in practice and does not significantly affect decoding performance.

Walk queries cannot be answered via Algorithm 4; instead, a localized graph search can be used for each matching root (*V*_*i*_, *o*_*i*_) of *Q* to reconstruct walks in the format $(o_{W_1},o_{W_K}):V_{W_1},...,V_{W_K}$, with offset $o_{W_0}$ denoting the start of the match in $V_{W_1}$ and offset $o_{W_K}$ the end of the match in $V_{W_K}$. Graph labels can be bit-encoded to support Walk queries efficiently. This adds to the flexibility of GIN-TONIC, while also allowing the integration of other indexing strategies (e.g. haplotype indexing) to optimize the search space of walks.

### Comparison with other tools

To pinpoint where GIN-TONIC lies in a complex landscape of indexing algorithms, in Table 3 we compare several state-of-the-art tools. While GBWT (41) indexes a collection of haplotypes stored as sequences of vertex identifiers, tools such as the r-index (43) and MOVI (44), based on (47) can be used to full-text index large collections of sequences, such as explicitly enumerated walks through a graph, but not graphs. Aligners allow for approximate searches within large collections of sequences but they do not store the full text, just part of it in the form of a set of minimizers. GCSA2 (25) indexes an approximate representation of the input graph. GIN-TONIC is the only approach listed in Table 3 that addresses the problem of exactly indexing graphs with an arbitrary topology and natively returning all matches for queries of any length.

As for searching functionality, the only method comparable to GIN-TONIC is GCSA2 (25). Given a string query, both tools aim to return walks in the graph containing the query. Unlike GIN-TONIC, however, GCSA2 approximates the input graph as a ‘pruned’ de Brujin graph with order up to some *k*-mer length determined at indexing time. GCSA2 may return false positives (but, according to what stated in (25), no false negatives) for queries >256 bp and constructs the approximate graph from the original one by computing paths. In contrast, GIN-TONIC supports queries of arbitrary length, can index graphs with arbitrary topology regardless of complexity, represents its input data exactly, does not need to enumerate paths, returns no false positives or negatives irrespective of query length, and, in addition, can compress the input data. Note that GIN-TONIC represents graphs as an abstraction of any data structure providing facilities similar to that of an FM-Index; hence, the r-index, MOVI, or other full-text indices might all be used as the underlying implementation of GIN-TONIC.

We set up the following experiment to compare the performance of GCSA2 to that of GIN-TONIC (fast). First, we used both tools to build indices for pangenome and transcriptome and measured construction times, peak memory usage, and index sizes. For the pangenome, we used as input the GFA file available in (38), constructed the graph via vg construct, and indexed the graph through vg index. As we were unable to get vg to directly process the transcriptome graph we had used as input in the section ‘Experiments and results’, we resorted to using vg rna instead, which can natively handle splicing graphs. To it we supplied the same annotations from which we had derived our transcriptome graph. We then generated for both graphs 1 million random queries of lengths 100, 256 and 1024 having at least one exact match along one of the graph paths. We measured the average time taken per query by GCSA2 and GIN-TONIC, as well as the time it took for each index to decode suffix array intervals (suffix array lookup part of Locate queries).

We report index construction statistics in Table 4. Compared to GCSA2, GIN-TONIC are significantly smaller. Furthermore, GIN-TONIC required roughly three orders of magnitude less time, and a fraction of the memory footprint of GCSA2, to build its indices. While we did not measure the disk space used by GCSA2, we remark that GIN-TONIC did not perform disk writes, as its indices can be constructed entirely in memory.

**Table 4.** Indexing benchmarks for GCSA2 and GIN-TONIC. Sizes are reported for both main index and auxiliary files (LCP for GCSA2; cache for GIN-TONIC)

Method	Size (GB)	LCP/Cache (GB)	CPU Time (min)	Mem (GB)
**Pangenome**				
**GCSA2**	7.6	5.6	9700	140
**GIN-TONIC**	3.0	1.3	12	39
**Transcriptome**				
**GCSA2**	8.7	5.6	15 000	187
**GIN-TONIC**	6.2	2.4	29	78

The results of querying benchmarks are reported in Table 5. In experiments involving the pangenome, both GIN-TONIC and GCSA2 found exact matches for all the simulated adversarial reads and real reads. Note that the results of the two tools are not directly comparable when it comes to the number of matching paths found— due to an implementation constraint, GCSA2 requires vertices to be split as no vertex label can be <32 bp. Therefore, before supplying the graph to GCSA2 to be indexed, $\texttt {vg}$ internally splits long sequences into smaller consecutive chunks of at most 32 bp. This makes the number of vertices and paths reported by GIN-TONIC and GCSA2 for the same query different, and hence such results cannot be directly compared. As for the transcriptome (entries marked with an asterisk (*) in Table 5), we observe strong differences, with GCSA2 never being able to recover all existing matches, and progressively less so as the read length increases. We suspect that such differences might be somehow due to an incorrect internal representation of the splice graph generated by $\texttt {vg rna}$. Excluding such dubious cases, the level of performance of the two tools is comparable, with GCSA2 being 2−3 times faster at Find queries and GIN-TONIC being 2−3 times faster at decoding matches. Of note, the results of GIN-TONIC could be further improved by using a more optimized implementation of its underlying FM-Index.

**Table 5.** Querying benchmarks for GCSA2 and GIN-TONIC over 1 million adversarially sampled queries for the pangenome and transcriptome, and actual short sequencing reads matching the graphs exactly (∼1.2 M for the pangenome, ∼2.0 M for the transcriptome). Times per Find query are reported, together with decoding times per query, and the number of patterns for which there were matches. The numbers marked with an asterisk (*) were obtained by using vg rna

Dataset	Pangenome			Transcriptome		
Query	Find	Dec.	Found	Find	Dec.	Found
Length	(μs)	(μs)		(μs)	(μs)	
**GIN-TONIC, 1 M adversarial reads**						
**100**	69	4	1 000 000	38	6	1 000 000
**256**	95	4	1 000 000	72	1	1 000 000
**1024**	202	4	1 000 000	221	1	1 000 000
**GCSA2, 1 M adversarial reads**						
**100**	22	12	1 000 000	32	8	919 800*
**256**	39	8	1 000 000	14	1	14 415*
**1024**	91	5	1 000 000	41	1	500*
**GIN-TONIC, real short reads**						
**95.8**	48	34	1 258 321	26	322	1 258 321
**GCSA2, real short reads**						
**72.3**	32	61	1 973 179	21	129	1 629 064*

As a final benchmark, in the Supplementary Material we also provide the results of querying the transcriptome with minigraph (see the section ‘Comparing minigraph and GIN-TONIC’ and Supplementary Table S5).

## Conclusions and outlook

In this paper we have introduced GIN-TONIC, a data structure able to index arbitrary string graphs non-hierarchically. In terms of construction times, memory usage and querying and decoding times, its performance approaches that of an FM-Index over linear text, as demonstrated by experimental measurements of Δ(*Q*) on datasets of the size of a human pangenome and transcriptome. We are not aware of any other work achieving such a result so far.

The experimental performance reported in the paper could be improved further in several ways—for instance, by parallelizing or pipelining several stages of the current implementation of GIN-TONIC, or by replacing its underlying FM-Index implementation with a hardware-optimized one. In addition, the representation of the string graph could be easily converted into a compressed and queryable file format and/or integrated into alignment software, possibly in conjunction with other haplotype indexing tools such as GBWT (19,20). Future work will also explore how more flexible querying strategies taking into account biological priors could be integrated into GIN-TONIC.

## Supplementary Material

lqae159_Supplemental_File

## Data Availability

Instructions and all mentioned input data compatible with GIN-TONIC (40) are available freely on https://github.com/uensalo/gin-data (permanent DOI: https://doi.org/10.5281/zenodo.10069416). Files used to construct the transcriptome are available at https://www.gencodegenes.org/human/release_40.html (39). The human pangenome (GRCh38-20-0.10b) is available via (38).
