# Supplementary material for: GIN-TONIC: non-hierarchical full-text indexing for graph genomes
Source: NAR Genom Bioinform. 2024 Dec 11;6(4):lqae159. doi: 10.1093/nargab/lqae159 (PMC11632618; doi:10.1093/nargab/lqae159)
Supplement: lqae159_Supplemental_File [file lqae159_supplemental_file.pdf]

Supplementary Material
GIN-TONIC: Non-hierarchical full-text indexing for graph-genomes

Unsal Ozturk[1], Marco Mattavelli[1], Paolo Ribeca[2,3,4,5]

[1]SCI-STI-MM, École Polytechnique Fédérale de Lausanne, Lausanne, Switzerland
[2]Biomathematics and Statistics Scotland, The James Hutton Institute, Edinburgh, United Kingdom
[3]Clinical and Emerging Infection, UK Health Security Agency, London, United Kingdom
[4]NIHR Health Protection Research Unit in Genomics and Enabling Data, University of Warwick, Coventry, United Kingdom
[5]NIHR Health Protection Research Unit in Gastrointestinal Infections, University of Liverpool, Liverpool, United Kingdom

(a)
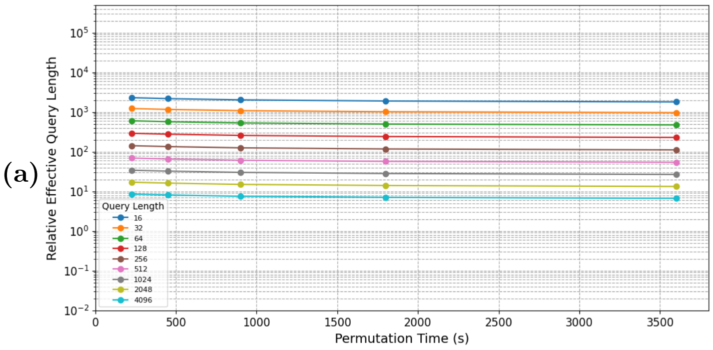

(b)
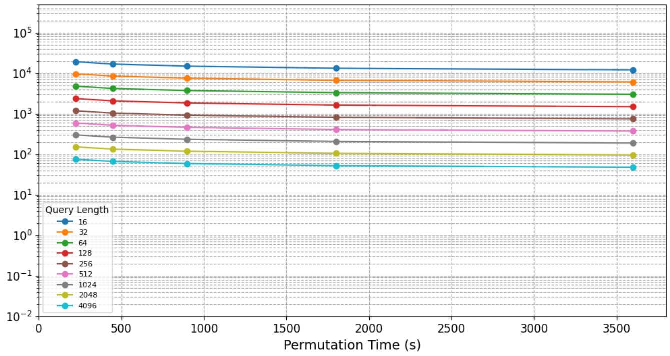

Supplementary Figure 1: Panels **(a,b)** show average $\Delta(Q)$ as a function of permutation optimization times for the pangenome (**a**) and the transcriptome (**b**).

(a)
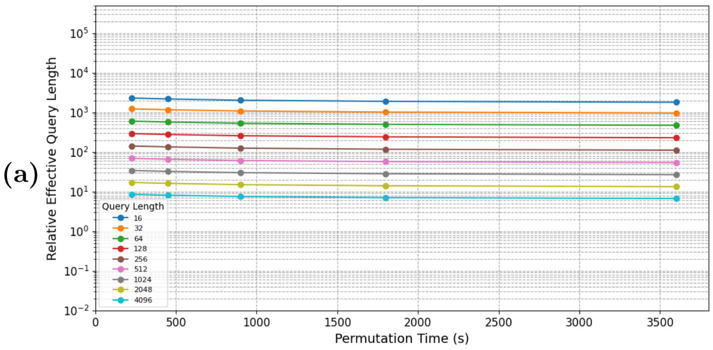

(b)
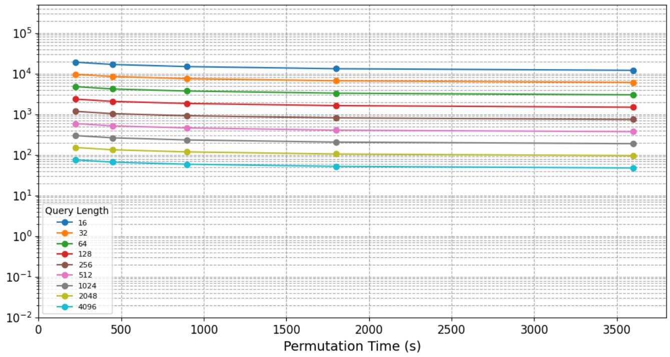

Supplementary Figure 2: Panels **(a,b)** show average $\Delta(Q)$ as a function of permutation optimization times for the pangenome (**a**) and the transcriptome (**b**).

Input Name	Pangenome	Transcriptome
**Size (MB)**	2,996.456	6,264.277
**|V|**	148,618	1,029,466
**|E|**	214,995	1,523,321
**Avg Label Length (bp)**	21,100	6,350
**Med Label Length (bp)**	265	161
**Std Label Length (bp)**	156,000	89,600
**Min Label Length (bp)**	1	1
**Max Label Length (bp)**	30,092,626	30,380,454
**Avg Indegree**	1.45	1.48
**Med Indegree**	1	1
**Std Indegree**	0.692	0.937
**Min Indegree**	1	1
**Max Indegree**	11	93
**Avg Outdegree**	1.45	1.48
**Med Outdegree**	1	1
**Std Outdegree**	0.689	0.873
**Min Outdegree**	1	1
**Max Outdegree**	8	71

Supplementary Table 1: Statistics for input items.

Cache Depth	Pangenome Size (MB)	Transcriptome Size (MB)
**1**	0.00	0.00
**2**	1.30	8.62
**3**	5.25	34.7
**4**	12.4	83.2
**5**	23.0	157
**6**	37.5	259
**7**	56.1	388
**8**	80.8	545
**9**	120	736
**10**	209	998
**11**	498	1,480
**12**	1,600	2,800

Supplementary Table 2: Cache sizes as a function of depth for both input items.

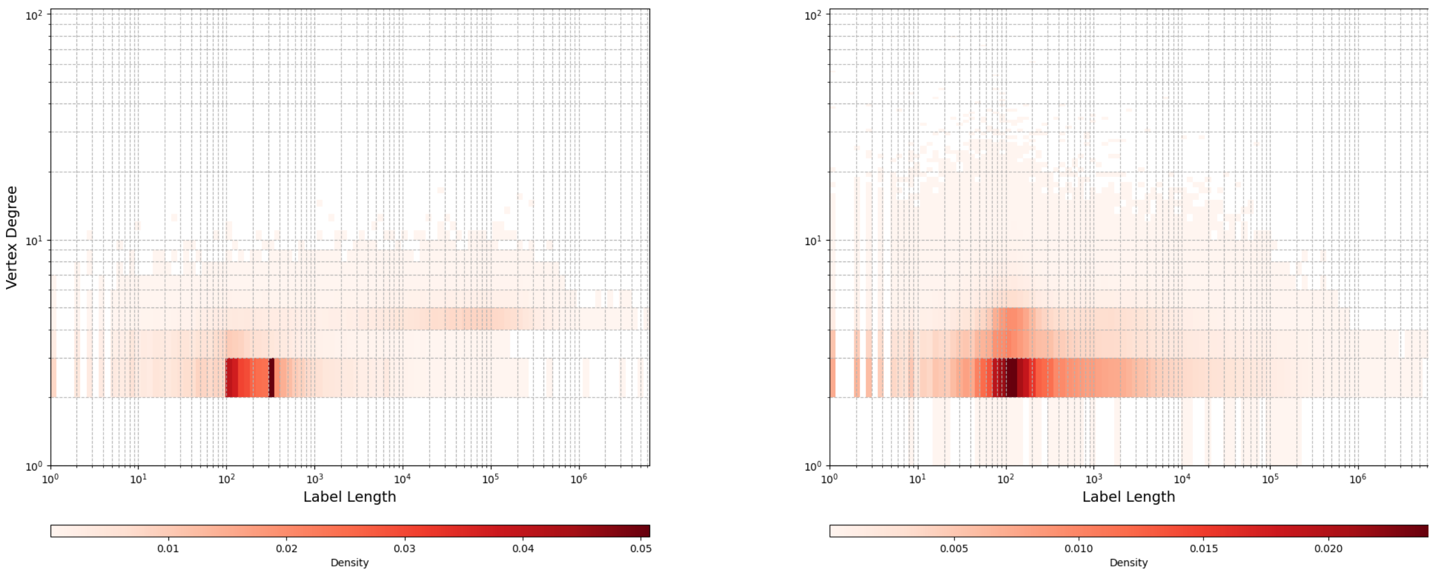

Supplementary Figure 3: Vertex label lengths vs. degrees for each vertex for pangenome (left) and transcriptome (right).

Asymptotic and Practical Time Complexity of GIN-TONIC-Lookup

The runtime complexity of the lookup can be characterised mainly by the number of ranges over the suffix array maintained in U at each iteration, which is initially populated with the suffix array range corresponding to the last character in the query (see line 3).

We now introduce simple bounds on the output size of R to obtain a worst-case bound on the number of states in U at a given iteration. The only way the number of ranges in U increases is through the introduction of new ranges through the evaluation of R (line 10), and the compaction of the ranges accumulated per state in U at the beginning of the while loop (line 5).

Lemma 1. Let $G(V, E, \mathbf{S})$ be a string graph and let $\mathrm{GE}(G, \Pi)$ be its graph encoding. The maximum number of suffix array ranges over the special character σ_0 returned by the range translator R satisfies

$$\max_{a,b \in \{1,\dots,|V|\}} |R([a,b])| = \left\lceil \frac{|V|}{2} \right\rceil$$

Proof. R is defined as

$$R([a,b]) = C_{\mathbb{I}}\left(\bigcup_{i=a}^{b} R_{\sigma_0}(i)\right)$$

i.e. the compaction of all suffix array ranges corresponding to each vertex represented in $[a, b]$. Note that the intervals returned by R, $[a_i, b_i]$ satisfy $a_i, b_i \in \{1, ..., |V|\}$, and that $a_{i+1} > b_i$ as the compaction $C_{\mathbb{I}}$ transforms a set of intervals into the union of those intervals expressed in terms of non-overlapping ones. The maximum number of non-overlapping intervals can be achieved by letting $[a_i, b_i] = [2i - 1, 2i - 1]$ for $i = 1, ..., \lceil |V|/2 \rceil$, hence resulting in $\lceil |V|/2 \rceil$ many ranges. To see why, add one more valid range $[x, x]$ not covered by $[a_i, b_i]$ to the set of returned intervals in an attempt to increase the number of ranges. Then, there exists some $[a_j, a_j]$ and $[a_{j+1}, a_{j+1}]$ such that $a_j < x < a_{j+1}$. Had $[x, x]$ been returned by the single range translator R_{σ_0}, then $[a_j, a_j]$, $[x, x]$, and $[a_{j+1}, a_{j+1}]$ would have been merged into $[a_j, a_{j+1}]$, hence resulting in $\lceil |V|/2 \rceil - 1$ ranges. Similarly, attempting to add a range $[x, y]$ overlapping with one or more $[a_i, a_i]$ would either decrease or not change the number of ranges returned. See Supplementary Figure 4. □

Corollary 1. The worst-case runtime complexity of Algorithm 1 occurs when the following are satisfied:

1. Each label $S_i \in \mathbb{S}$ is a single character σ.
2. $G(V, E, \mathbb{S})$ is a bipartite graph $G(U_1 \cup U_2, E, \mathbb{S}_{U_1} \cup \mathbb{S}_{U_2})$ where U_1, U_2 are the two parts of the bipartite graph, and $\mathbb{S}_{U_1} \cup \mathbb{S}_{U_2}$ are the string labels associated with each part.
3. $||U_1| - |U_2|| \le 1$.

Algorithm 1 GIN-TONIC Lookup

procedure GIN-TONIC-LOOKUP$(Q, (\mathrm{I}_{\mathrm{FMI}}, R, r_{\sigma_0}))$
  $[d_a, d_b) \leftarrow$ ADVANCE-RANGE$([0, |\mathrm{GE}(G, \Pi)|), Q[|Q|-1], \mathrm{I}_{\mathrm{FMI}})$
  $U \leftarrow \{[d_a, d_b)\}$
  $t \leftarrow |Q| - 2$
  while $t \geq 0$ and $|U| > 0$ **do**
    $F \leftarrow \{\ \}$
    for $[d_a, d_b) \in U$ **do**
      $[v_a, v_b) \leftarrow$ ADVANCE-RANGE$([d_a, d_b), \sigma_0, \mathrm{I}_{\mathrm{FMI}})$
      if $v_b > v_a$ **then**
        $I_{List} \leftarrow R([v_a, v_b - 1])$
        for $[v_s, v_e]$ in I_{List} **do**
          $F \leftarrow F \cup [v_s, v_e + 1)$
        end for
      end if
    end for
    $F \leftarrow$ COMPACT-INTERVALS(F)
    $U^{'} \leftarrow \{\ \}$
    for $[d_a, d_b) \in U \cup F$ **do**
      $[n_a, n_b) \leftarrow$ ADVANCE-RANGE$([d_a, d_b), Q[t], \mathrm{I}_{\mathrm{FMI}})$
      if $n_b > n_a$ **then**
        $U^{'} \leftarrow U^{'} \cup [n_a, n_b)$
      end if
    end for
    $U \leftarrow U^{'}$
    $t \leftarrow t - 1$
  end while
  return SUFFIX-ARRAY-DECODE$(U, (\mathrm{I}_{\mathrm{FMI}}, r_{\sigma_0}))$
end procedure

Supplementary Figure 4: Maximum suffix array occupancy of the ranges returned by R. First line: optimal suffix array occupancy of returned ranges. $|V|$ is not coloured if it is even. Second line: Attempting to insert one more range always causes a compaction of the adjacent ranges, hence reducing the number of ranges by one.

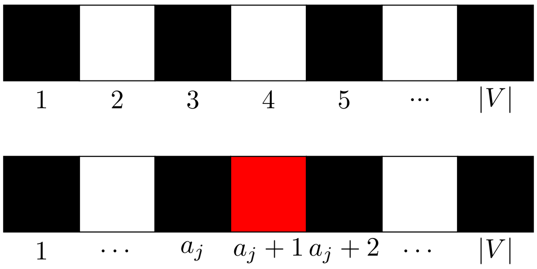

4. Π interleaves vertices from different parts, i.e. for each $i \in [1, |V| - 1]$, either $V_i \in U_1$ and $V_{i+1} \in U_2$ or $V_i \in U_2$ and $V_{i+1} \in U_1$.

5. $Q = \sigma^n$ for some positive n.

These conditions essentially 1. force each evaluation of R to return $\lceil |V|/2 \rceil$ states for each state in U at the start of the loop; 2. force the output size of Compact-Intervals at line 16 to be $\lceil |V|/2 \rceil$ while maximising the size of F, and 3. make sure that no states are discarded by forcing the if statement at line 20 to always evaluate to true. The interleaved permutation enforces the states to be merged into $\lceil |V|/2 \rceil$ states, which is the maximum number of states achievable after a merge.

Given the conditions imposed by the graph described in Corollary 1, one can prove that the number of states are polynomially bounded by the number of iterations, which itself is bounded by the length of the query.

Lemma 2. Given a query Q consisting on the same character σ, U contains $|Q| \lceil |V|/2 \rceil + 1$ ranges at line 27 for a GIN-TONIC constructed on the graph G described in Corollary 1.

Proof. Define iteration $i = 0$ to be the state of U before any characters are processed, and let U contain a single range, the range over all characters as in line 2, in this iteration. $i > 0$, iteration i could be defined as the ith time the loop starting at line 5 is executed. Let f_i denote the size of F before line 16 is executed at iteration i, and similarly let c_i be the size of F after line 16 is executed, and s_i be the size of U after line 24 is executed. One has the following tail recursive relations between the aforementioned quantities for $i > 0$:

$$\begin{aligned} f_i &= \lceil |V|/2 \rceil \, s_{i-1} \\ c_i &= \lceil |V|/2 \rceil \\ s_i &= s_{i-1} + c_i \end{aligned}$$

with $f_0 = 0$, $c_0 = 0$, $s_0 = 1$. The expression for f_i is a result of the call to R in line 10 producing $\lceil |V|/2 \rceil$ by Lemma 4 per input state due to the topology G and the interleaved permutation Π. Due to the same occupancy principle in Lemma 1, c_i can be at most $\lceil |V|/2 \rceil$, and due to Π is equal to $\lceil |V|/2 \rceil$ in this case. The expression for s_i is simply due the union in line 18, and that no ranges are filtered out since there is always a match by condition 1 in Corollary 1. Hence, summing, one has

$$\begin{aligned} \sum_{j=1}^{i} s_j &= \sum_{j=1}^{i} (s_{j-1} + c_i) \\ s_i &= s_0 + i \lceil |V|/2 \rceil \\ &= i \lceil |V|/2 \rceil + 1 \end{aligned}$$

Let $i = |Q|$ to obtain the expression for $s_{|Q|}$. □

Now that we have a bound for the number of states at a given iteration, we can compute an expression for the computational cost of a single iteration i.

Theorem 1. Algorithm 1 has worst-case polynomial runtime.

Proof. Assume that branching, list insertions and unions, memory accesses, and arithmetic operations take constant time. Let $w(i)$ denote the number of operations performed at iteration i. Denote upper bound on the cost of evaluating R as W_R, the upper bound on compacting k intervals as $w_C(k)$, and the cost of advancing range as W_A. Assume that the indexed graph has topology a described in Corollary 1, and that the permutation is interleaved. We write

$$w(i) = \underbrace{(W_A + W_R)\left((i-1)\left\lceil\frac{|V|}{2}\right\rceil + 1\right)}_{\text{1: Forking Phase}} + \underbrace{w_C\left(\left\lceil\frac{|V|}{2}\right\rceil\left((i-1)\left\lceil\frac{|V|}{2}\right\rceil + 1\right)\right)}_{\text{2: Compaction Phase}} + \underbrace{W_A\left(i\left\lceil\frac{|V|}{2}\right\rceil + 1\right)}_{\text{3: Advance Phase}}$$

as the total cost incurred during the ith iteration. The first term counts the total number of times Advance-Range is called and R is evaluated per state in U. The second term is for the compaction, where the size of the input to Compact-Intervals is equal to the number of ranges obtained by evaluating R per range in U. The third term is the number of times Advance-Range is called for the states in $S \cup F$. Summing from 1 to $|Q|$ yields the total cost $W(i)$:

$$W(i) = \sum_{i=1}^{|Q|} \left((W_A + W_R)\left((i-1)\left\lceil \frac{|V|}{2} \right\rceil + 1\right) + w_C\left(\left\lceil \frac{|V|}{2} \right\rceil \left((i-1)\left\lceil \frac{|V|}{2} \right\rceil + 1\right)\right) + W_A\left(i\left\lceil \frac{|V|}{2} \right\rceil + 1\right)\right)$$

$$= \sum_{i=1}^{|Q|} \left((W_A + W_R)\left((i-1)\left\lceil \frac{|V|}{2} \right\rceil + 1\right)\right) + \sum_{i=1}^{|Q|} \left(w_C\left(\left\lceil \frac{|V|}{2} \right\rceil \left((i-1)\left\lceil \frac{|V|}{2} \right\rceil + 1\right)\right)\right) + \sum_{i=1}^{|Q|} \left(W_A\left(i\left\lceil \frac{|V|}{2} \right\rceil + 1\right)\right)$$

For the forking phase one has:

$$\begin{aligned}
&\sum_{i=1}^{|Q|} \left((W_A + W_R)\left((i-1)\left\lceil \frac{|V|}{2} \right\rceil + 1\right)\right) \\
&= (W_A + W_R)\left(|Q| + \left\lceil \frac{|V|}{2} \right\rceil \sum_{i=1}^{|Q|}(i-1)\right) \\
&= (W_A + W_R)\left(|Q| + \frac{1}{2}\left\lceil \frac{|V|}{2} \right\rceil |Q|(|Q|-1)\right)
\end{aligned}$$

For the advance phase:

$$\begin{aligned}
&\sum_{i=1}^{|Q|} \left(W_A\left(i\left\lceil \frac{|V|}{2} \right\rceil + 1\right)\right) \\
&= W_A\left(|Q| + \left\lceil \frac{|V|}{2} \right\rceil \sum_{i=1}^{|Q|} i\right) \\
&= W_A\left(|Q| + \frac{1}{2}\left\lceil \frac{|V|}{2} \right\rceil |Q|(|Q|+1)\right)
\end{aligned}$$

For the compaction phase, one needs to determine $w_C(.)$, i.e. the number of operations performed per interval processed, which depends on the implementation. If R returns sorted intervals, $w_C(.)$ can be implemented via a min-heap: the problem boils down to merging $(i-1)\left\lceil \frac{|V|}{2} \right\rceil + 1$ intervals, each of which has size $\left\lceil \frac{|V|}{2} \right\rceil$ then merging a list of sorted intervals. If this recipe is followed, w_C at iteration i needs on the order of $\left\lceil \frac{|V|}{2} \right\rceil \left((i-1)\left\lceil \frac{|V|}{2} \right\rceil + 1\right) \log\left((i-1)\left\lceil \frac{|V|}{2} \right\rceil + 1\right)$ operations. Summing from 1 to $|Q|$, one obtains the total cost:

$$\begin{aligned}
&\sum_{i=1}^{|Q|} \left(\left\lceil \frac{|V|}{2} \right\rceil \left((i-1)\left\lceil \frac{|V|}{2} \right\rceil + 1\right) \log\left((i-1)\left\lceil \frac{|V|}{2} \right\rceil + 1\right)\right) \\
&= \left\lceil \frac{|V|}{2} \right\rceil \left(\sum_{i=1}^{|Q|} \left((i-1)\left\lceil \frac{|V|}{2} \right\rceil + 1\right) \log\left((i-1)\left\lceil \frac{|V|}{2} \right\rceil + 1\right)\right)
\end{aligned}$$

The sum can be bounded from above by the integral of the summand with bounds 1 to $|Q|+1$, i.e.,

$$\begin{aligned}
B &= \int_1^{|Q|+1} \left((x-1)\left\lceil \frac{|V|}{2} \right\rceil + 1\right) \log\left((x-1)\left\lceil \frac{|V|}{2} \right\rceil + 1\right) dx \\
&\geq \sum_{i=1}^{|Q|} \left((i-1)\left\lceil \frac{|V|}{2} \right\rceil + 1\right) \log\left((i-1)\left\lceil \frac{|V|}{2} \right\rceil + 1\right)
\end{aligned}$$

The integral can be computed as a closed-form expression by integrating by parts, yielding

$$B = \frac{|Q|^2 \left\lceil \frac{|V|}{2} \right\rceil \log\left(|Q|\left\lceil \frac{|V|}{2} \right\rceil + 1\right)}{2} - \frac{|Q|^2 \left\lceil \frac{|V|}{2} \right\rceil}{4} + |Q| \log\left(|Q|\left\lceil \frac{|V|}{2} \right\rceil + 1\right) - \frac{|Q|}{2} + \frac{\log\left(|Q|\left\lceil \frac{|V|}{2} \right\rceil + 1\right)}{2\left\lceil \frac{|V|}{2} \right\rceil}$$

with $B \in O(|Q|^2|V|\log(|Q||V|))$. Hence the compaction phase has a complexity of $O(|Q|^2|V|^2\log(|Q||V|))$.

Finally, we make an assumption on the number of operations performed by W_A and W_R. ADVANCE-RANGE can be implemented in constant time through a bitvector supporting constant time rank and get operations. R can be represented in many ways: one could cache all precomputed incoming neighbours in the suffix array domain for any range over $|V|$ vertices, which yields a constant time operation with quadratic space requirements in $|V|$. Another option is to merge intervals on the fly, resulting in quadratic time. A middle ground is to implement an interval-merge tree, which is a middle ground between caching and on-the-fly merging. Assuming that all merges are looked up from a precomputed table, i.e. W_R is constant, the number of operations for the forking phase and the advance phase is $O(|Q|^2|V|)$, and is $O(|Q|^2|V|^2\log(|Q||V|))$ for the compaction phase. The overall complexity of Algorithm 1 is hence $O(|Q|^2|V|^2\log(|Q||V|))$, i.e. it is dominated by the compaction. □

Note that the bound $O(|Q|^2|V|^2\log(|Q||V|))$ is obtained under the adversarial assumptions given under Corollary 1. The performance of Algorithm 1 under typical use cases, where the number of incoming neighbours in the input graphs are bounded by a constant, tends to be empirically better. With a cache, as discussed in the main paper, it is possible to achieve performance close to that of an FM-index over linear text.

Effect of Permutations

Supplementary Figures 1, 2 show the relation between the time spent optimising the permutation and the relative effective query length $\Delta(Q)$, which measures the slowdown introduced by the topology of the graph with respect to the linear FM-Index. No cache was used here, as the speed-ups provided by the two mechanisms are not independent. While the permutation can make the index more efficient, its influence is relatively modest with respect to that of the cache. It's also worth noting that even though optimising the permutation does improve querying efficiency, it also incurs a one-off time cost at indexing time. In our case, we optimised the permutation for an hour at most, which is comparable to the time to build the FM-Index itself.

Scalability Tests with Multi-Indexing

We tested the scalability of GIN-TONIC to larger pangenomic graphs (in terms of number of graph elements and incorporated genomic variants) and benchmarked GIN-TONIC on the HPRC pangenome available under `https://github.com/human-pangenomics/hpp_pangenome_resources`. Further information about how this pangenome was obtained is available on the given link. The pangenome file `hprc-v1.1-mc-grch38.gfa` contains 80,069,733 segments, 110,938,345 links, 25,770 walks indicating haplotypes, and a sum of 3,234,968,488 bp of nucleotides. The total size of the file comes is 48.1GB; however, as the GIN-TONIC toolbox does not index haplotypes and only indexes graph elements, we filter out the lines containing haplotypes and end up with only the pangenomic graph - this results in a 9GB GFA file. We then convert the bidirected representation of the pangenomic graph into an equivalent directed representation. In particular, we parse `L` lines whose orientations are not (`+`, `+`), and then explicitly create vertices containing the reverse complement of the sequence of the segment of the line marked on the reverse complement, and generate directed edges accordingly.

To index the resulting graph in a time and space efficient manner, we first separate it into smaller subgraphs where each subgraph is a connected component of the input file. This results in 179 distinct graph components, 23 of which correspond to chromosome graph and the rest correspond to other components. We then partition the file into 24 smaller graphs: one per chromosome and one for other components into a 24th graph file. The distribution of segments, nucleotides, and links in each of these partitions is given in Supplementary Table 3.

Each partition is then a) indexed independently via GIN-TONIC and b) suffix-array cached for $\Sigma^{\leq 12}$, using 24 processes in parallel in indexing and 8 processes in parallel while caching. Overall, the indexing task took 256 seconds in wall-clock time (24 processes at a time), and the caching took 1981 seconds (8 processes at a time). The relevant statistics are given in Supplementary Table 4, and are plotted as a bar chart in Supplementary Figure 5.

As for the querying performance of the multi-index, we generated 1 million queries of length 100bp sampled randomly from the original graph file. In the querying phase, all of the caches and indices were loaded into memory over 24 GIN-TONIC query instances running concurrently. A program receives queries and broadcasts all of these queries to each index, and each index writes its results to its temporary file, which are then appropriately concatenated into a single file once the querying is over. For each partition, we gather relevant performance statistics regarding querying: the index loading time, the cache loading time, and "slack time" spent cleaning up and deallocating memory once the querying is over. Supplementary Figure 6 depicts the querying times and the performance of individual indices over each partition. The querying speed supported by this multi-index is determined by the longest querying time among all partitions, which occurred in partition 9 with 188 seconds. The multi-index took 188μs per query, 1.88μs per character overall, and computed 10,139,365,441 walk roots

**Parition**	**Segments**	**Nucleotides**	**Links**
1	3,408,312	137,139,560	4,733,247
2	6,450,773	249,816,123	8,924,099
3	3,506,987	142,212,849	4,852,230
4	2,342,523	106,586,502	3,244,645
5	4,983,257	204,057,517	6,888,314
6	1,859,947	67,661,698	2,574,969
7	2,097,024	67,119,364	2,932,711
8	5,304,202	196,842,987	7,332,383
9	6,323,654	265,043,440	8,740,153
10	4,517,930	175,798,190	6,258,690
11	4,623,766	170,349,632	6,400,147
12	3,537,605	139,541,833	4,892,403
13	2,151,620	83,133,388	2,975,606
14	1,304,541	48,587,824	1,817,516
15	4,985,241	191,300,862	6,933,402
16	2,784,694	95,931,420	3,850,191
17	2,604,312	117,271,150	3,603,941
18	4,135,502	153,950,818	5,708,615
19	2,420,493	110,413,881	3,362,217
20	2,334,393	88,434,354	3,253,724
21	3,061,656	160,780,100	4,273,568
22	3,794,653	139,028,134	5,252,967
23	1,279,333	54,036,663	1,780,739
24	257,315	69,930,199	351,868

Supplementary Table 3: Distribution of segments, nucleotides, and links of the partitions of the initial GFA file `hprc-v1.1-mc-grch38.gfa`

Partition	Index Size (MB)	Indexing Time (seconds)	Cache Size
1	355.16	118.14	3340M
2	695.38	235.83	5612M
3	362.66	117.95	3458M
4	275.99	86.91	2537M
5	529.80	163.76	4471M
6	181.76	62.17	2176M
7	194.13	58.01	2390M
8	553.47	172.70	4735M
9	667.61	221.33	5494M
10	500.52	152.19	4162M
11	492.82	147.95	4262M
12	385.50	123.22	3428M
13	215.79	63.64	2424M
14	131.88	27.94	1780M
15	504.71	161.23	6650M
16	286.36	91.48	2955M
17	308.12	96.01	2734M
18	441.48	137.83	3933M
19	282.76	89.38	3469M
20	235.70	69.31	2498M
21	354.84	111.27	3088M
22	380.54	124.29	3652M
23	143.12	33.63	1734M
24	85.45	9.16	803M

Supplementary Table 4: Index sizes, indexing times, and suffix array cache sizes for each partition of the HPRC pangenome.

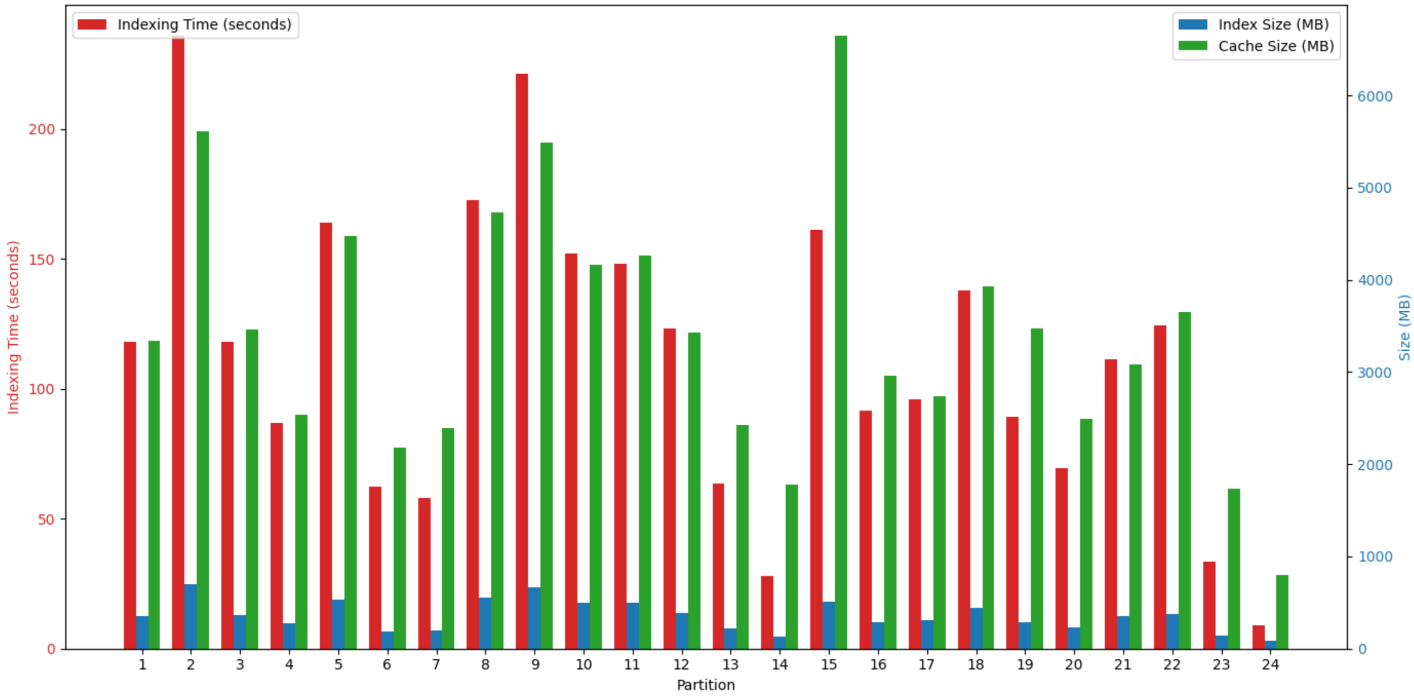

Supplementary Figure 5: Bar chart of index sizes, indexing times, and suffix array cache sizes for each partition of the HPRC pangenome.

for 1,000,000 queries in total, and the peak memory usage was 199GB, which includes all indices, caches, and partial or unwritten query results.

Another interesting observation is that the querying complexity of the multi-index was highly correlated with the number of links contained within the partition: The Spearman correlation coefficient between querying speeds and the number of links was computed to be 0.993043. This suggests that the querying time for the multi-index can be further improved by further partitioning the partition that takes the longest time into smaller graphs incrementally at the cost of increasing the number of concurrent indices. In summary, designing multi-indices are a challenge on their own, and present many trade-offs between computational resources and querying efficiency. Nonetheless, the approach presented in this document demonstrates that GIN-TONIC can scale to graphs larger in the number of graph components, i.e. vertices and edges.

Comparing `minigraph` and GIN-TONIC

In addition to GCSA2, we also ran experiments `minigraph`. We set up the following experiment: We translated both the forward and the reverse strands of the transcriptome to GFA format, and tried to align adversarial exact string queries generated off of the transcriptome using `minigraph`. These were the same string queries (of length 16,32,64,...,4096) used throughout the benchmarks for GIN-TONIC. There were 65536 string queries of each length.

Query Length	Exact Alignments Found	Inexact Alignments Found
16	0	0
32	0	0
64	0	14,568
128	0	45,488
256	0	62,866
1024	0	65,536
2048	0	65,536
4096	0	65,536

Supplementary Table 5: Query lengths, the number of exact alignments found, and the number of inexact alignments found for 65536 adversarial queries over the transcriptome via `minigraph`.

As each query is simulated exactly from the input transcriptome, the ground truth of the alignment mapping contains

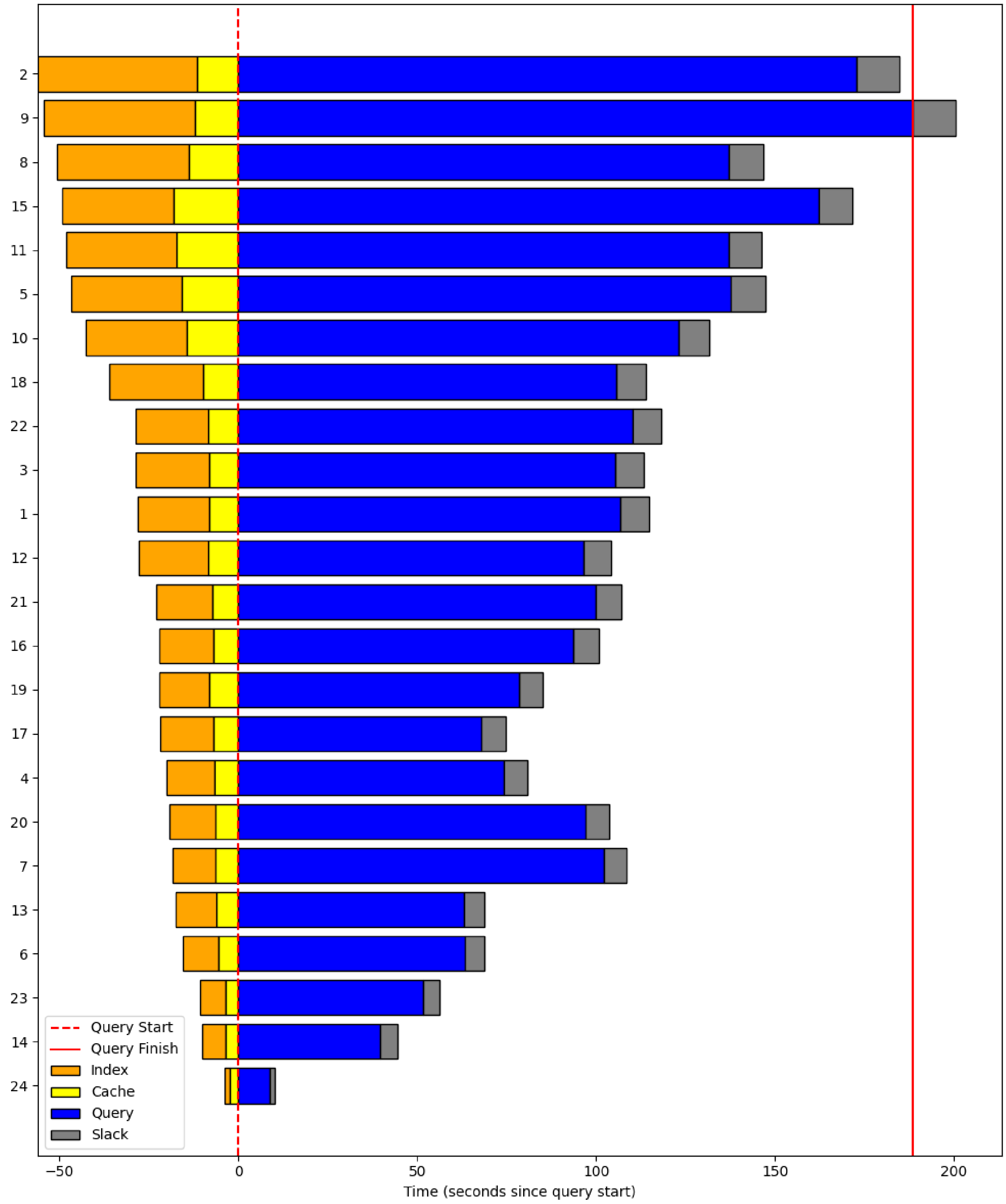

Supplementary Figure 6: Gannt chart for GIN-TONIC multi-process querying over 24 partitions (indices do not necessarily correspond to chromosome numbers) of the HPRC pangenome. The partitions are sorted with respect to the number of links they contain. Index refers to index loading times, cache refers to cache loading times, and query refers to the actual time spent on computing queries.

at least one perfect match or exact alignment for each query. We inspected the output of `minigraph` for each query file and counted the number of exact alignments per query, and we could not find any perfect alignments of the simulated queries to the transcriptome in the output files. The reason why query lengths 16,32 have no alignments output for them is due to `minigraph`'s anchoring and chaining heuristic: the queries are too short for anchors belonging to them to be chained. For longer queries, the program did output alignments; however, none of them were exact and we expect at least one alignment per query to be exact. This is presumably due to the target application of `minigraph`'s heuristics of minimiser-based anchoring and chaining. These results suggest that the areas of application and the use cases for `minigraph` and GIN-TONIC are different, and the two tools may not be directly compared: GIN-TONIC is a graph index that supports only exact queries, and `minigraph` is a minimizer-based graph aligner.
